# Denitrification and Nitrate-Dependent Fe(II) Oxidation in Various *Pseudogulbenkiania* Strains

**DOI:** 10.1264/jsme2.ME16001

**Published:** 2016-07-15

**Authors:** Satoshi Ishii, Kazuki Joikai, Shigeto Otsuka, Keishi Senoo, Satoshi Okabe

**Affiliations:** 1 Department of Soil, Water, and Climate; BioTechnology Institute, University of Minnesota140 Gortner Laboratory, 1479 Gortner Ave., St. Paul, MN 55108–6106USA; 2 Division of Environmental Engineering, Faculty of Engineering, Hokkaido UniversityKita 13, Nishi 8, Kita-ku, Sapporo, Hokkaido 060–8628Japan; 3 Department of Applied Biological Chemistry, The University of Tokyo1–1–1 Yayoi, Bunkyo-ku, Tokyo 113–8657Japan

**Keywords:** *Pseudogulbenkiania*, nitrate-dependent Fe(II) oxidation, denitrification, mutagenesis, comparative genomics

## Abstract

*Pseudogulbenkiania* is a relatively recently characterized genus within the order *Neisseriales*, class *Betaproteobacteria*. This genus contains several strains that are capable of anaerobic, nitrate-dependent Fe(II) oxidation (NDFO), a geochemically important reaction for nitrogen and iron cycles. In the present study, we examined denitrification functional gene diversities within this genus, and clarified whether other *Pseudogulbenkiania* sp. strains perform denitrification and NDFO. Seventy strains were analyzed, including two type strains, a well-characterized NDFO strain, and 67 denitrifying strains isolated from various rice paddy fields and rice-soybean rotation fields in Japan. We also attempted to identify the genes responsible for NDFO by mutagenesis. Our comprehensive analysis showed that all *Pseudogulbenkiania* strains tested performed denitrification and NDFO; however, we were unable to obtain NDFO-deficient denitrifying mutants in our mutagenesis experiment. This result suggests that Fe(II) oxidation in these strains is not enzymatic, but is caused by reactive N-species that are formed during nitrate reduction. Based on the results of the comparative genome analysis among *Pseudogulbenkiania* sp. strains, we identified low sequence similarity within the *nos* gene as well as different gene arrangements within the *nos* gene cluster, suggesting that *nos* genes were horizontally transferred. Since *Pseudogulbenkiania* sp. strains have been isolated from various locations around the world, their denitrification and NDFO abilities may contribute significantly to nitrogen and iron biogeochemical cycles.

The genus *Pseudogulbenkiania*, which belongs to the family *Chromobacteriaceae* (1), order *Neisseriales*, class *Betaproteobacteria*, was first proposed by Lin *et al.* (27). Two species have since been reported: *Pseudogulbenkiania subflava* isolated from a cold spring in Taiwan (27) and *Pseudogulbenkiania gefcensis* isolated from soil in South Korea (26).

The genus *Pseudogulbenkiania* also contains the anaerobic, nitrate-dependent Fe(II)-oxidizing bacterium strain 2002 (also called “*Pseudogulbenkiania ferrooxidans*”), isolated from a freshwater lake in Illinois, USA (42, 44), and strain MAI-1, isolated from a freshwater lake in Indonesia (23). Anaerobic, nitrate-dependent Fe(II) oxidation (NDFO) is a process in which ferrous iron (Fe[II]) is oxidized to ferric iron (Fe[II]) coupled with the reduction of nitrate under anoxic, circumneutral conditions (5, 9, 38, 43, 45). This reaction is ecologically important (28, 29) and has great potential for biotechnological applications such as the bioremediation of toxic metals (11, 19, 24, 32, 33). However, Fe(II) oxidoreductase has not yet been identified among NDFO microbes (6), which limits our understanding of this geochemically important biological reaction. Since some *Pseudogulbenkiania* strains have the potential to be genetically engineered (23), their use is advantageous for the study of NDFO.

We previously isolated 67 *Pseudogulbenkiania* strains by using a functional single-cell isolation method (3) from rice paddy fields and rice-soybean rotation fields in Kumamoto, Niigata, and Yamagata in Japan (39). These strains showed strong denitrification and N_2_O reduction activities. The findings of a culture-independent RNA-based analysis also suggest that *Pseudogulbenkiania* spp. strongly contribute to denitrification and N_2_O reduction in rice paddy soils (46). Rice paddy fields are abundant in nitrate and Fe(II) (12, 16, 35) and NDFO activity has also been detected (12, 16, 35); therefore, *Pseudogulbenkiania* spp. may be involved in NDFO in the environment. However, the NDFO ability of *Pseudogulbenkiania* denitrifiers isolated from rice paddy soils has not yet been examined. Furthermore, relatedness among the *Pseudogulbenkiania* denitrifiers, NDFO strains 2002 and MAI-1, and other *Pseudogulbenkiania* species has not been analyzed to date. We targeted denitrification functional genes (nitrite reductase gene [*nirS*] and nitrous oxide reductase gene [*nosZ*]) in order to analyze diversity among strains.

The objectives of the present study are (i) to examine the NDFO abilities of *Pseudogulbenkiania* strains, (ii) to identify the genes responsible for NDFO, and (iii) to analyze denitrification functional gene diversities within the genus *Pseudogulbenkiania*.

## Materials and Methods

### Bacterial strains

Sixty-seven *Pseudogulbenkiania* strains were previously isolated from rice paddy fields and rice-soybean rotation fields in Kumamoto (29 strains), Niigata (33 strains), and Yamagata (5 strains) in Japan (39). Among these strains, *Pseudogulbenkiania* sp. strain NH8B was selected as a representative strain to sequence its whole genome (14). *P. subflava* strain BP-5^T^ (= LMG 24211^T^), *P. gefcensis* strain yH16^T^ (= JCM 17850^T^), and *Pseudogulbenkiania* sp. strain 2002 (= ATCC BAA-1479) were obtained from the Belgian Coordinated Collections of Microorganisms (BCCM), Japan Collection of Microorganisms (JCM), and American Type Culture Collection (ATCC), respectively. Bacterial cells were maintained in R2A medium (Wako Pure Chemical) at 30°C.

The plasmid vector pRL27, which contains a hyperactive Tn5 transposase gene (25), was used in transposon mutagenesis. *Escherichia coli* WM3064, a 2,6-diaminopimelic acid (DAP) auxotroph, was used as a donor organism for the pRL27 vector (20, 37). This strain was maintained in LB agar medium supplemented with DAP (300 μg mL^−1^) and kanamycin (100 μg mL^−1^).

### Iron oxidation assays

Standard anaerobic culturing techniques with anoxic grove box (Coy laboratories) with an N_2_:CO_2_:H_2_ (80:10:10) atmosphere were used for this experiment. Cells grown on R2A agar were suspended in anoxic basal medium supplemented with 5 mM nitrate in test tubes with butyl rubber stoppers. Basal medium contained the following chemicals (L^−1^): 0.25 g of NH_4_Cl, 0.6 g of NaH_2_PO_4_, 0.1 g of KCl, 2.52 g of NaHCO_3_, and 10 mL each of the trace metal solution and vitamin solution (45). The cells were then incubated under anoxic conditions (N_2_:CO_2_:H_2_ = 80:10:10) at 30°C. This incubation was performed in order to deplete carbon in the cells. After a 3-d incubation, cells were pelleted and washed three times with anoxic PIPES (piperazine-*N*,*N*′-bis[2-ethanesulfonic acid]) buffer (10 mM, pH 7.0). Washed cells (final conc. 5 × 10^7^ cells mL^−1^ as established by microscopy and OD_600_ measurements [15]) were inoculated into anoxic basal medium (20 mL) supplemented with 10 mM FeCl_2_ and 5 mM NaNO_3_ in test tubes with butyl rubber stoppers. After replacing the air phase of the test tubes with H_2_-free anoxic gas (N_2_:CO_2_ = 80:20), cells were incubated at 30°C. Samples (2 mL) were periodically collected using syringe needles. Aliquots (100 μL) of the samples were immediately mixed with 700 μL of 1 M HCl to stabilize Fe(II) and dissolve Fe(II) minerals. Twenty microliters was kept frozen at −20°C for a quantitative PCR (qPCR) analysis (see below). The remainders of the samples (*ca.* 1.9 mL) were filtered through 0.20-μm pore membrane filters and stored at −20°C until used for ion chromatography.

Fe(II) concentrations were measured spectrophotometrically using the ferrozine method as described by Hegler *et al.* (10). In order to measure total Fe concentrations (Fe[II] + Fe[III]), Fe(III) was reduced by 50% (w/v in 1M HCl) hydroxylamine hydrochloride prior to measurements by the ferrozine method. Nitrate and nitrite concentrations were measured using an ion chromatograph IC-2010 equipped with the TSKgel SuperIC-Anion HS column (Tosoh).

A high-throughput iron oxidation assay was performed using 96-well plates. In brief, cells grown on R2A agar were suspended in anoxic basal medium (150 μL) supplemented with 5 mM nitrate. After a 3-d anoxic incubation, cells (15 μL) were transferred to anoxic basal medium (135 μL) supplemented with 10 mM FeCl_2_ and 5 mM NaNO_3_, and incubated under anoxic conditions (N_2_:CO_2_:H_2_ = 80:10:10) at 30°C. Before and after a 1-week incubation, 25 μL of the cell culture was mixed with 175 μL of 1 M HCl solution in order to measure Fe(II) and total Fe concentrations, as described above. *Pseudogulbenkiania* sp. strain 2002 was used as a positive control.

### Testing for autotrophy by NDFO

In an attempt to clarify whether cells have the ability to grow under NDFO conditions, we performed quantitative PCR by using strain-specific primers (IAC_23F and IAC_92R primers; Table S1) (15) and the KOD Sybr qPCR Mix (Toyobo). Culture medium (1 μL) in the NDFO assay was directly used as a template for qPCR. Standard DNA was prepared by diluting the linearized plasmid (15) with basal medium containing the same concentrations of potential PCR inhibitors (*i.e.*, Fe^2+^) as the samples. The amplification efficiency (*E*) of the assay was 76% with a linear dynamic range from 20–2 × 10^6^ copies μL^−1^.

We also performed stable isotope probing using ^13^C-labeled bicarbonate. Cells were prepared as described above, and incubated in anoxic basal medium (20 mL) supplemented with 10 mM FeCl_2_ and 5 mM NaNO_3_. All bicarbonate in this medium was labeled with ^13^C (Cambridge isotope laboratories). As a control, cells were incubated in medium with ^12^C bicarbonate. DNA was extracted after a 9-d incubation by using the PowerSoil DNA Isolation Kit (MoBio Laboratories). Cesium chloride density gradient ultracentrifugation and DNA collection were performed as previously described (13, 30). DNA concentrations in each of the density-resolved fractions were quantified using the Quant-iT PicoGreen ds DNA Assay Kit (ThermoFisher Scientific).

### Transposon mutagenesis

Conjugation was performed as described by Kim *et al.* (20). In brief, *E. coli* pRL27 donor strain WM3064 and the recipient *Pseudogulbenkiania* sp. strain NH8B were mixed at a 1:4 ratio, and spotted on R2A agar supplemented with DAP (300 μg mL^−1^). After an overnight incubation, cells were suspended in R2A broth and spread onto R2A agar supplemented with kanamycin (100 μg mL^−1^). Colonies grown on this agar were selected and tested for their iron-oxidizing ability, as described above.

### DNA extraction, PCR, and Sequencing

DNA was extracted by heating cells in 100 μL of 0.05 M NaOH at 95°C for 15 min (3). After centrifugation, the supernatant was diluted 10-fold and used for PCR as described below.

The 16S rRNA gene and *nirS* were amplified using m-27F and m-1492R primers (41) and m-cd3aF and m-R3cd primers (18), respectively. Regarding the amplification of *nosZ*, we slightly modified the sequences of the nosZ-F-1181 and nosZ-R-1880 primers (36). The nosZ-F-1181_NH8B and nosZ-R-1880_NH8B primers and nosZ-F-1181_2002 and nosZ-R-1880_2002 primers (Table S1) allow for the amplification of *nosZ* from strains NH8B and 2002, respectively. In addition, in order to amplify the region between *nosZ* (locus tag NH8B_3640) and the gene encoding the NosZ-like protein (locus tag NH8B_3641) of strain NH8B, PCR was performed using nosZ-R-1880_NH8B and IAC_92R (15) (Table S1).

The PCR reaction mixture (50 μL) contained 1 × *Ex Taq* buffer (Takara Bio), 0.2 μM of each primer, 0.2 mM of each dNTP, 1 U of *Ex Taq* DNA polymerase (Takara Bio), and 1 μL of a DNA template. PCR was performed using a Veriti 96-well thermal cycler (Applied Biosystems) with the following conditions: initial annealing at 96°C for 5 min, followed by 35 cycles at 95°C for 30 s, the annealing temperature shown in Table S1 for 30 s, and 72°C for the extension time shown in Table S1. After the final extension at 72°C for 7 min, the PCR mixtures were stored at 4°C. The sizes of the PCR products were verified by agarose gel electrophoresis.

PCR products were purified using the FastGene Gel/PCR Purification Kit (Nippon Genetics) and sequenced using the BigDye Terminator v3.1 Cycle Sequencing Kit (Applied Biosystems) and ABI 3730*xl* capillary sequencer (Applied Biosystems) with the primers shown in Table S1 and Fig. S1.

### Transcription analysis

*Pseudogulbenkiania* sp. strain 2002 and strain NH8B were grown in basal medium (5 mL) supplemented with 10 mM acetate under oxic or anoxic conditions. When grown under anoxic conditions, 5 mM nitrate was added to this medium as an electron acceptor. Total RNA was extracted using the RiboPure Bacteria kit (Ambion). After the DNase treatment, complementary DNA (cDNA) was synthesized using random hexamers and a PrimeScript RT reagent kit (Perfect Real Time) (Takara Bio). The resulting cDNA was used to detect gene transcription by PCR (Table S2). While genomic DNA was used as a positive control, RNA samples were used as negative controls in order to verify the absence of contamination by genomic DNA. Experiments were performed in triplicate (*i.e.*, three test tubes per condition for each strain).

### Genomic and phylogenetic analyses

Multiple reads obtained by sequencing reactions were assembled using the Phred-Phrap program (7). The nucleotide sequences of multiple clones were aligned using ClustalW, and used to generate phylogenetic trees. Phylogenetic trees were constructed based on the maximum likelihood method by using MEGA ver. 6 (40).

The genome of *Pseudogulbenkiania* sp. strain NH8B was previously sequenced (14). The genome sequences of *Pseudogulbenkiania* sp. strain 2002 (4) and *Chromobacterium violaceum* ATCC 12472 were retrieved from the GenBank database. These genomes were compared using GenomeMatcher (31).

### Nucleotide sequence accession numbers

The nucleotide sequences of the 16S rRNA gene, *nirS*, and *nosZ* were deposited in the DDBJ/EMBL/GenBank databases under the accession numbers KU175358–KU175423, KU175424–KU175493, and KU175494–KU175563, respectively (Table S3).

## Results and Discussion

### Anaerobic nitrate-dependent Fe(II) oxidation

**Fig. 1:**
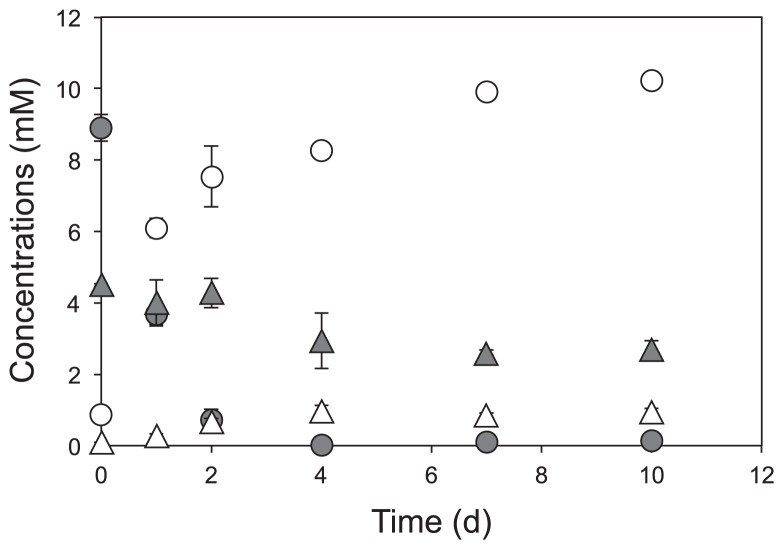
Occurrence of anaerobic, nitrate-dependent Fe(II) oxidation by *Pseudogulbenkiania* sp. strain NH8B. Washed cells (5 × 10^7^ cells) were inoculated into anoxic basal medium supplemented with 10 mM FeCl_2_ and 5 mM NaNO_3_, and incubated under anoxic conditions at 30°C for 10 d. Legends: Concentrations of Fe(II) ( 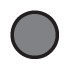), Fe(III) (○), NO_3_^−^ ( 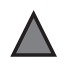), and NO_2_^−^ (△). The mean ± SD is shown.

*Pseudogulbenkiania* sp. strain NH8B exhibited NDFO activity: Fe(II) was rapidly oxidized to Fe(III) along with the reduction of nitrate to nitrite (Fig. 1), similar to previous findings obtained with *Pseudogulbenkiania* sp. strain 2002 (42, 44). In contrast, the oxidation of Fe(II) was not observed when no cells or heat-killed cells were added to the test tubes (data not shown). These results indicate that Fe(II) was oxidized as a result of biological activity, either by a direct or indirect reaction.

The amount of nitrate consumed (ΔNO_3_^−^ = −1.89 ± 0.08 mM; Table S4) in the 7-d incubation was larger than the amount of nitrite produced (ΔNO_2_^−^ = 0.76 ± 0.08 mM), indicating that nitrite was further reduced to gaseous nitrogen oxides and dinitrogen. Based on the electron balance of the NDFO reaction (eq.1 and 2), two and five moles of Fe(II) are required to reduce one mole of nitrate to nitrite and to N_2_ gas, respectively.

(eq. 1) $$2{\text{Fe}}^{2+}+{{\text{NO}}_{3}}^{-}+2{\text{H}}^{+}\to 2{\text{Fe}}^{3+}+{{\text{NO}}_{2}}^{-}+{\text{H}}_{2}\text{O}$$

(eq. 2) $$5{\text{Fe}}^{2+}+{{\text{NO}}_{3}}^{-}+6{\text{H}}^{+}\to 5{\text{Fe}}^{3+}+1/2{\text{N}}_{2}+3{\text{H}}_{2}\text{O}$$

If 0.76 mM and 1.13 mM (= 1.89 – 0.76 [mM]) of nitrate were reduced to nitrite and N_2_ gas, respectively, 1.52 mM and 5.65 mM of Fe^2+^ were oxidized according to eq. 1 and 2, respectively. The amount of Fe(II) oxidized in this study (ΔFe^2+^ = −8.78 ± 0.37 mM) was larger than the theoretical amount of Fe(II) required to reduce nitrate to nitrite and N_2_ gas (7.17 mM) as calculated above. This result suggested the occurrence of the abiotic oxidation of Fe(II) by reactive nitrogen species (*e.g.*, nitrous acid [HNO_2_], nitrogen dioxide [NO_2_], and nitric oxide [NO]) formed during the acidic Fe extraction step (eq. 3–7) (21).

(eq. 3) $${{\text{NO}}_{2}}^{-}+{\text{H}}^{+}\to {\text{HNO}}_{2}$$

(eq. 4) $$2{\text{HNO}}_{2}\to {\text{NO}}_{2}+\text{NO}+{\text{H}}_{2}\text{O}$$

(eq. 5) $$2{\text{Fe}}^{2+}+{\text{NO}}_{2}+2{\text{H}}^{+}\to 2{\text{Fe}}^{3+}+\text{NO}+{\text{H}}_{2}\text{O}$$

(eq. 6) $${\text{Fe}}^{2+}+2\text{NO}+{\text{H}}^{+}\to {\text{Fe}}^{3+}+\text{HNO}$$

(eq. 7) $$2\text{HNO}\to {\text{N}}_{2}\text{O}+{\text{H}}_{2}\text{O}$$

(eq. 8) $$2\text{NO}+{\text{O}}_{2}\to 2{\text{NO}}_{2}$$

The reactions shown in eq. 3–7 proceed abiotically, particularly under acidic conditions (*e.g.*, during the HCl extraction of Fe minerals) (21). Furthermore, NO may be oxidized back to NO_2_ under oxic conditions (eq. 8). Although our HCl extraction was performed in the anoxic grove box, the ferrozine assay was conducted under a 20% O_2_ atmosphere. Thus, some Fe(II) may have been abiotically oxidized during sample processing. This may partly explain the discrepancy between the rates of Fe(II) oxidation and nitrate reduction: *i.e.*, most Fe(II) was oxidized within the first 2 d, while nitrate was reduced for a longer time period (*ca.* 4–7 d) in Fig. 1. The accumulation of nitrite may be due to the depletion of electron donors (*e.g.*, Fe[II] and organic substrates stored in cells).

Cell growth was not observed during the incubation period (Fig. S2). In addition, ^13^C-bicarbonate was not incorporated into nucleic acids based on the SIP analysis (Fig. S3), suggesting that NDFO does not support the autotrophic growth of strain NH8B. Thus, NDFO-dependent growth may differ according to the strains (33, 38, 44) or test conditions.

The oxidation of Fe(II) was also observed in all other *Pseudogulbenkiania* sp. strains based on our high-throughput iron oxidation assay (Table S3), suggesting that NDFO is a common characteristic among *Pseudogulbenkiania* sp. strains.

In order to identify the genes associated with Fe(II) oxidoreductase, we performed transposon mutagenesis using strain NH8B. A total of 1,440 mutants were obtained and screened based on NDFO activity. As expected, mutants unable to grow under anoxic conditions on R2A agar supplemented with nitrate did not exhibit NDFO activity. We were unable to obtain mutants that did not oxidize iron, but had the ability to grow by anaerobic nitrate reduction, indicating that mutants disrupted with Fe(II) oxidoreductase-associated genes were not obtained in this study. In addition, the genome of strain NH8B did not contain homologs to *mtrAB* and *pioAB*, which are involved in respiratory iron reduction and phototrophic iron oxidation, respectively (17, 34). The homologs to *mtrAB* and *pioAB* are candidates for a potential Fe(II) oxidoreductase gene.

Similar to the present study, a previous attempt to identify specific Fe(II) oxidoreductase in the NDFO strain, *Acidovorax ebreus* strain TPSY, using a proteomic approach was not successful (6). These authors showed that respiratory complexes such as nitrate reductase (Nar) directly mediated NDFO, indicating that a specific Fe(II) oxidoreductase is not necessarily required for NDFO (6, 24). In addition, biotically or abiotically formed NO_2_ or NO may also react with Fe(II) to form Fe(III) oxides (6, 21, 22). These results suggest that NDFO exhibits an intrinsic ability in all nitrate-reducing bacteria, and may explain the failure to obtain the mutants of interest (*i.e.*, NDFO-incapable, nitrate-reducing mutants). Strong NDFO abilities among *Pseudogulbenkiania* spp. may depend on their toxicity tolerance of Fe(II) and nitrogen oxides (*e.g.*, NO_2_^−^ and NO), the extent of the accumulation of nitrogen oxides, and/or the nature of their respiratory proteins (6).

### Genome comparison

Genome structures and gene contents were similar between *Pseudogulbenkiania* sp. strains NH8B and 2002 (Fig. S4A), although there were several inverted genome fragments. In contrast, genome structures differed between *Pseudogulbenkiania* sp. strains NH8B and *C. violaceum* ATCC 12472 (Fig. S4B), a strain phylogenetically close to *Pseudogulbenkiania* sp. strains (Fig. S5).

**Fig. 2:**
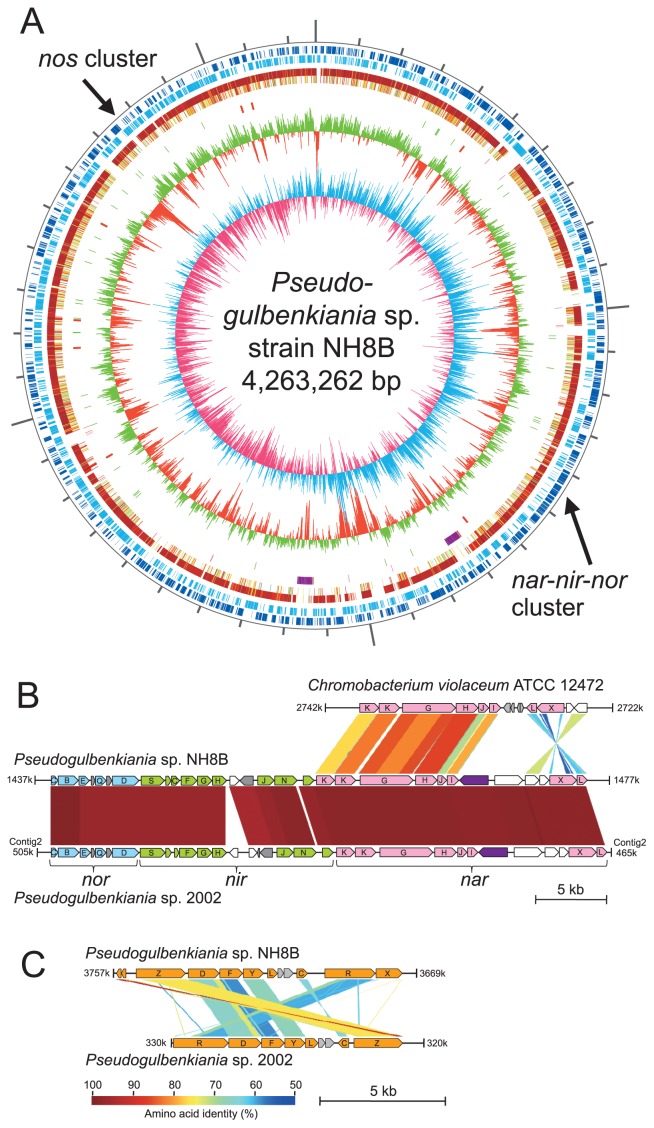
Comparison of genomes of *Pseudogulbenkiania* sp. strain NH8B, strain 2002, and *Chromobacterium violaceum* ATCC 12472. (A) Circular representation of the *Pseudogulbenkiania* sp. NH8B genome. From the outside in: circles 1 and 2 of the chromosome show the positions of protein-coding sequences on the positive and negative strands, respectively. Circles 3 and 4 show the positions of protein-coding sequences that have orthologs in *Pseudogulbenkiania* sp. 2002 and *Chromobacterium violaceum* ATCC 12472, respectively. Circle 5 shows the positions of the prophages (purple) and integrative elements (pink). Circle 6 shows the positions of tRNA genes (green) and rRNA genes (red). Circle 7 shows a plot of the G + C content (higher values outward). Circle 8 shows a plot of the GC skew ([G − C]/[G + C]; light blue indicates values > 0; red indicates values < 0). (B) Schematic diagram of nitrate reductase (Nar), nitrite reductase (Nir), and nitric oxide reductase (Nor) gene clusters from strain NH8B, strain 2002, and *C. violaceum* ATCC 12472. (C) Schematic diagram of the nitrous oxide reductase (Nos) gene cluster from strain NH8B and strain 2002.

Complete sets of denitrification functional genes were identified on the genomes of the strains NH8B and 2002. Strains NH8B and 2002 both possessed gene clusters for membrane-bound Nar, cytochrome *cd*_1_-containing nitrite reductase (Nir), nitric oxide reductase (Nor), and nitrous oxide reductase (Nos). While *nar*-*nir*-*nor* operons were located next to each other, the *nos* operon was distantly located on the genome in strains NH8B and 2002 (Fig. 2A).

The gene arrangement within the *nar*-*nir*-*nor* cluster was similar between strains NH8B and 2002 (Fig. 2B). In addition, gene sequences were highly conserved between the two strains. For example, the *nirS* sequence was 98.6% similar between strain NH8B and strain 2002. In contrast, the gene arrangement within the *nos* cluster was different between the two strains, and *nos* gene sequence similarities were relatively low (Fig. 2C). These results suggest that one of the two *nos* clusters was horizontally acquired. Further studies (*e.g.*, comparative genome analysis with many other strains) may be necessary in order to obtain concrete evidence of the horizontal gene transfer event.

Interestingly, strain NH8B possessed 183-bp-long *nosZ*like pseudogenes (locus tag NH8B_3641 and NH8B_3642) downstream of the full-length *nosZ* (locus tag NH8B_RS17660), which were 84% similar to the *nosZ* of strain 2002.

### *nirS* and *nosZ* diversity

In order to further investigate *nirS* and *nosZ* sequence diversities within the genus *Pseudogulbenkiania*, we sequenced *nirS* and *nosZ* from 70 *Pseudogulbenkiania* sp. strains (*i.e.*, 67 *Pseudogulbenkiania* sp. strains isolated in our previous study, *Pseudogulbenkiania* sp. strain 2002, *P. subflava* strain BP-5^T^, and *P. gefcensis* strain yH16^T^). Since commonly used PCR primers for *nosZ* (nosZ-F-1181 and nosZ-R-1880) did not amplify *nosZ* from strains NH8B and 2002, we designed new primers.

The *nirS* sequences from all *Pseudogulbenkiania* sp. strains were relatively highly conserved among the 70 strains, with >90.4% similarity (367 bp). *Pseudogulbenkiania* sp. strains isolated from different locations were clustered together based on their *nirS* sequences (Fig. S6).

**Fig. 3:**
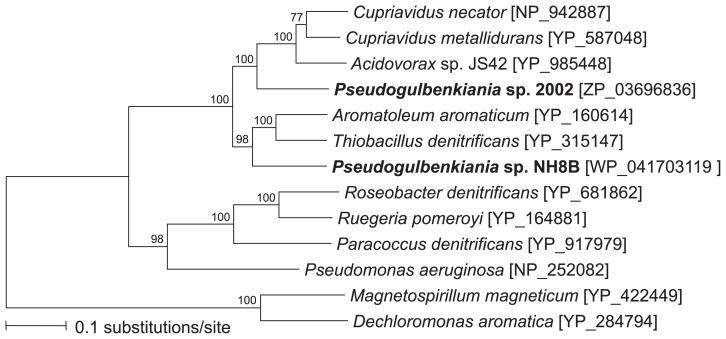
Phylogenetic relationships of deduced NosZ amino acid sequences between *Pseudogulbenkiania* sp. strain NH8B and *Pseudogulbenkiania* sp. strain 2002. A phylogenetic tree was constructed using the maximum likelihood method. Bootstrap values (>70%) from 1,000 replicates are indicated next to the branches. The accession numbers of the reference strains in the DDBJ/EMBL/GenBank databases are indicated in the brackets.

In contrast, we observed greater dissimilarity among *nosZ* from *Pseudogulbenkiania* sp. strains (> 59.4% sequence similarities among 633-bp fragments). Based on *nosZ* sequence similarities, the 70 *Pseudogulbenkiania* sp. strains were divided into two groups: NH8B-type (15 strains) and 2002-type (55 strains) groups (Fig. 3).

All 15 NH8B-type strains had a *nosZ*-like pseudogene similar to strain NH8B, based on NH8B_3641-specific qPCR (15) and an analysis of the sequences downstream of *nosZ*. The intergenic region between *nosZ* and the *nosZ*-like pseudogene was relatively well conserved among the 15 strains (Fig. S7). The intergenic region is known to be generally more variable than protein-coding sequences (8). The highly conserved intergenic regions among the 15 strains indicate that these strains may have diversified relatively recently from a common ancestor, and were then distributed to different locations.

In this region, we detected a FNR-binding motif (FNR box; TTGAT----ATCAA), which is recognized by FNR-like transcription regulators and reported to be essential for the transcription of *nosZ* in *Pseudomonas aeruginosa* (2). We detected the transcription of *nosZ* and *nosZ*-like pseudogenes in the cells of *Pseudogulbenkiania* sp. strain NH8B under oxic and anoxic conditions (Fig. S8A and S8B). In contrast, the transcription of *nosZ* was only detected in the cells of *Pseudogulbenkiania* sp. strain 2002 (Fig. S8C) grown under anoxic conditions, suggesting that transcription regulation may differ between strains NH8B and 2002. The role of FNR-like transcription regulators in the transcription of *nosZ* and related proteins is currently unknown. Since *Pseudogulbenkiania* sp. strain NH8B reduces exogenous N_2_O (14), a deeper understanding of the transcription mechanism of *nosZ* will be useful for mitigating N_2_O emissions.

## Conclusions

Our comprehensive analysis showed that diverse strains within the genus *Pseudogulbenkiania* perform denitrification and NDFO. The failure to detect NDFO-deficient denitrifying mutants in our mutagenesis experiment suggests that NDFO is linked to nitrate-reducing activity (*i.e.*, abiotic Fe[II] oxidation by reactive nitrogen species formed during nitrate reduction) without the involvement of specific Fe(II) oxidoreductase. The NDFO abilities of *Pseudogulbenkiania* spp. may depend on their toxicity tolerance of Fe(II) and nitrogen oxides (*e.g.*, NO_2_^−^ and NO), the extent of the accumulation of nitrogen oxides, and/or the nature of their respiratory proteins (6). Different gene arrangements within the *nos* cluster and low sequence similarity within *nos* gene sequences among *Pseudogulbenkiania* sp. strains indicate the occurrence of horizontal gene transfer. Since *Pseudogulbenkiania* sp. strains have been isolated from various locations (*e.g.*, agricultural soils and freshwater lakes) around the world, their denitrification and NDFO abilities may contribute significantly to nitrogen and iron biogeochemical cycles.

## Supplementary Information
