# Supplementary material for: Denitrification and Nitrate-Dependent Fe(II) Oxidation in Various Pseudogulbenkiania Strains
Source: Microbes Environ. 2016 Jul 15;31(3):293–8. doi: 10.1264/jsme2.ME16001 (PMC5017806; doi:10.1264/jsme2.ME16001)
Supplement: Supplementary file 1 [file 31_293_s1.pdf]

Supplementary Information

Denitrification and Nitrate-Dependent Fe(II)-Oxidation in Various Pseudogulbenkiania Strains

Satoshi Ishii, Kazuki Jokai, Shigeto Otsuka, Keishi Senoo, and Satoshi Okabe

Table S1. PCR primers used in this study.

Target gene	Primer name	Primer sequence (5'-->3')	Annealing temp. (°C)	Extension time (sec)	Reference
16S rRNA gene	m-27F	AGRGTTTGATYMTGGCTCAG	55	90	Tyson *et al*. 2004
	m-1492R	GGYTACCTTGTTACGACTT			
nirS	m-cd3aF	AACGYSAAGGARACSGG	58*	60	Kandeler *et al*. 2006
	m-R3cd	GASTTCGGRTGSGTCTTSAYGAA			
nosZ (NH8B type)	nosZ-F-1181_NH8B	CGCTCTTCCTGGATAGCCAG	60	60	This study
	nosZ-R-1880_NH8B	ATGCAGGGCATGGCAGAA			
	NH8B_3826510F	TTCAATCCACAAGCCAAACAG	60	30	This study
	NH8B_3827082R	TATTGCACCAACTTCTGCCA			
nosZ (2002 type)	nosZ-F-1181_2002	CGCTGTTCCTCGACTCGCAG	60	60	This study
	nosZ-R-1880_2002	AGRTGCAGSGCRTGGCAGAA			
	2002_323853F	GCCAGTATTTCGCCGTAGTC	60	30	This study
	2002_323846R	ATGTCCTACCAGAACTTCTTCC			
Intergenic region between *nosZ* and *nosZ*-like pseudogene	nosZ-R-1880_NH8B	ATGCAGGGCATGGCAGAA	56	120	This study
	IAC_92R	GAGGCGATGTGGATGGTC			Ishii *et al*. 2013
	NH8B_3828317F	AATTCGCTACCGCAGAACAC	60	30	This study
	NH8B_3829464R	GGTCATCTTGTTGCTGTCCA			

*Toutchdoun PCR from 63°C (-1°C/cycle)

Table S2. Sequencing primers used in this study.

Target gene	Primer name	Primer sequence (5"-->3")	Reference
Intergenic region between *nosZ* and *nosZ*-like pseudogene	NH8B_3827573F	AGGAAGAATTTGCCATCCGGT	This study
	NH8B_3828165F	TTGATTGGAGCAGGTCAGCT	This study
	NH8B_3828945F	CCAAGTCAGGATTCAGAGATT	This study
	NH8B_3828925R	AATCTCTGAATCCTGACTTGG	This study

Table S3. The NDFO activity of the strains as examined by the high-throughput iron oxidation assay. Proportion of Fe(II) oxidized was calculated as a function of Fe(III) divided by the total amount of Fe in the medium after 1-week incubation. The GenBank accession numbers to the nucleotide sequences of the 16S rRNA gene, *nirS*, and *nosZ* are also shown.

Strain ID	Proportion of Fe(II) oxidized (%)	GenBank Accession Number		
		16S rRNA gene	*nirS*	*nosZ*
KH1B	100.8	KU175358	KU175424	KU175494
KH2C	84.3	KU175359	KU175425	KU175549
KH3E	92.5	KU175360	KU175426	KU175495
KH4DB	99.8	KU175361	KU175427	KU175496
KH5A	66.4	KU175362	KU175428	KU175497
KH6C	90.8	KU175363	KU175429	KU175498
KH7B	92.8	KU175364	KU175430	KU175550
KH10D	100.7	KU175365	KU175431	KU175551
KH26C	66.8	KU175366	KU175432	KU175499
KH32A	42.6	KU175367	KU175433	KU175500
KH34	80.6	KU175368	KU175434	KU175501
KH39B	99.7	KU175369	KU175435	KU175502
KH40B	48.2	KU175370	KU175436	KU175503
KH41A	94.3	KU175371	KU175437	KU175504
KH43A	66.4	KU175372	KU175438	KU175505
KH48C	85.6	KU175373	KU175439	KU175506
KS1B	79.1	KU175374	KU175440	KU175507
KS3B	58.7	KU175375	KU175441	KU175508
KS8A	80.1	KU175376	KU175442	KU175509
KS11C	85.8	KU175377	KU175443	KU175510
KS14A	100.2	KU175378	KU175444	KU175511
KS30B	71.5	KU175379	KU175445	KU175512
KS32B	37.0	KU175380	KU175446	KU175552
KS33D	40.2	KU175381	KU175447	KU175513
KS35C	100.4	KU175382	KU175448	KU175514
KS42A	41.6	KU175383	KU175449	KU175515
KS43AB	54.5	KU175384	KU175450	KU175516
KS45A	101.0	KU175385	KU175451	KU175517
KS46A	101.1	KU175386	KU175452	KU175518
NH2B	82.0	KU175387	KU175453	KU175553
NH4	55.5	KU175388	KU175454	KU175554
NH7	100.3	KU175389	KU175455	KU175519
NH8B	100.4	NR_074657	KU175456	KU175555
NH11	65.6	KU175390	KU175457	KU175520
NH15	100.9	KU175391	KU175458	KU175521
NH16	54.5	KU175392	KU175459	KU175522
NH19	71.7	KU175393	KU175460	KU175556
NH27B	100.1	KU175394	KU175461	KU175523
NH30A	90.8	KU175395	KU175462	KU175557
NH33B	83.9	KU175396	KU175463	KU175558
NH34	87.1	KU175397	KU175464	KU175559
NH38B	99.7	KU175398	KU175465	KU175524
NH48C	89.7	KU175399	KU175466	KU175560
NH49	67.8	KU175400	KU175467	KU175561

Table S3. Continued

Strain ID	Proportion of Fe(II) oxidized (%)	GenBank Accession Number		
		16S rRNA gene	*nirS*	*nosZ*
NS6	72.1	KU175401	KU175468	KU175525
NS11	65.9	KU175402	KU175469	KU175526
NS12B	55.0	KU175403	KU175470	KU175527
NS13E	53.3	KU175404	KU175471	KU175528
NS15	100.2	KU175405	KU175472	KU175529
NS20B	41.5	KU175406	KU175473	KU175530
NS21B	99.3	KU175407	KU175474	KU175531
NS22	100.8	KU175408	KU175475	KU175532
NS23	63.1	KU175409	KU175476	KU175533
NS24EB	52.6	KU175410	KU175477	KU175534
NS25	67.5	KU175411	KU175478	KU175535
NS27A	46.9	KU175412	KU175479	KU175536
NS28	62.9	KU175413	KU175480	KU175537
NS31A	81.4	KU175414	KU175481	KU175538
NS38B	99.5	KU175415	KU175482	KU175539
NS39	70.9	KU175416	KU175483	KU175540
NS41	51.2	KU175417	KU175484	KU175541
NS46	58.9	KU175418	KU175485	KU175542
YNH5A	88.2	KU175419	KU175486	KU175543
YNH10AA	92.2	KU175420	KU175487	KU175544
YNH19CA	100.9	KU175421	KU175488	KU175545
YNH28A	100.5	KU175422	KU175489	KU175562
YNH30A	85.3	KU175423	KU175490	KU175563
2002	82.1	AY609199	KU175491	KU175546
LMG 24211	68.8	NR_044327	KU175492	KU175548
JCM 17850	98.7	NR_118145	KU175493	KU175547

Table S4. Concentrations [mM] of NO_3^-, NO_2^-, Fe(II), and Fe(III) in the anoxic basal medium 0 day and 7 days after inoculation of *Pseudogulbenkiania* sp. strain NH8B. The same data were used to draw Figure 1.

	NO_3^-					NO_2^-				
	A	B	C	Mean	SD	A	B	C	Mean	SD
Day 1	4.47	4.54	4.47	4.50	0.04	0.08	0.09	0.09	0.09	0.00
Day 7	2.68	2.60	2.54	2.60	0.07	0.89	0.76	0.90	0.85	0.07
Delta	-1.80	-1.94	-1.94	-1.89	0.08	0.81	0.67	0.81	0.76	0.08

	Fe(II)					Fe(III)				
	A	B	C	Mean	SD	A	B	C	Mean	SD
Day 1	9.29	8.55	8.86	8.90	0.37	0.69	0.97	0.95	0.87	0.16
Day 7	0.13	0.13	0.12	0.12	0.00	9.73	9.93	10.05	9.90	0.16
Delta	-9.17	-8.42	-8.74	-8.78	0.37	9.04	8.96	9.10	9.03	0.07

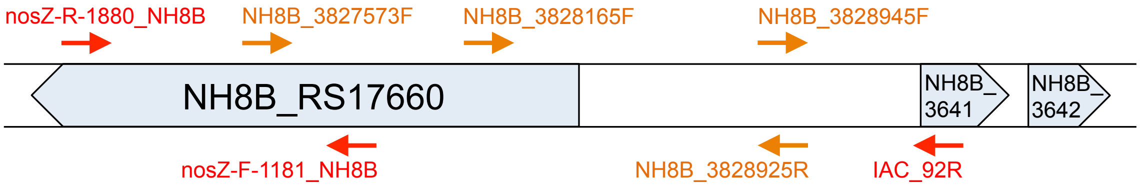

Figure S1. Annealing sites of the primers used to amplify and sequence *nosZ* and the intergenic region between *nosZ* (NH8B_RS17660) and *nosZ*-like pseudogene (NH8B_3641 and NH8B_3642). Red and brown arrows indicate the annealing sites of the primers for PCR and sequencing reactions, respectively. Primer sequences are shown in Table S1.

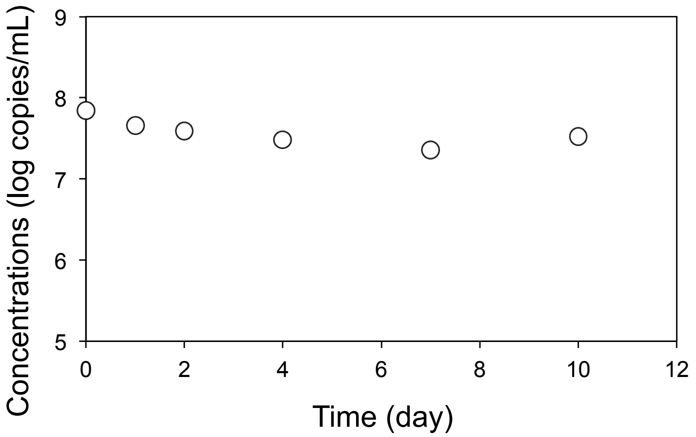

Figure S2. Concentration of cells in the culture medium, as measured by strain-specific quantitative PCR. Cells were collected from the medium in the NDFO conditions (Fig. 1). Target molecule is present in one copy/genome; therefore, copies/mL is equivalent to cells/mL.

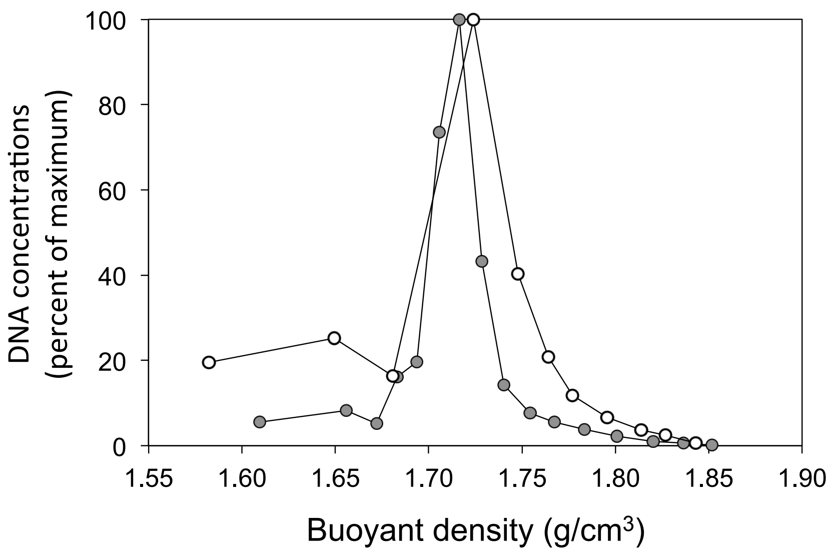

Figure S3. Cesium chloride density gradient centrifugation of DNA extracted from cells. Legends: ●, cells incubated in the medium containing ^{13}C-labeled bicarbonate, 5 mM nitrate, and 10 mM $FeCl_2$; ○, cells incubated in the medium containing ^{13}C-labeled bicarbonate, 5 mM nitrate, and 10 mM $FeCl_2$.

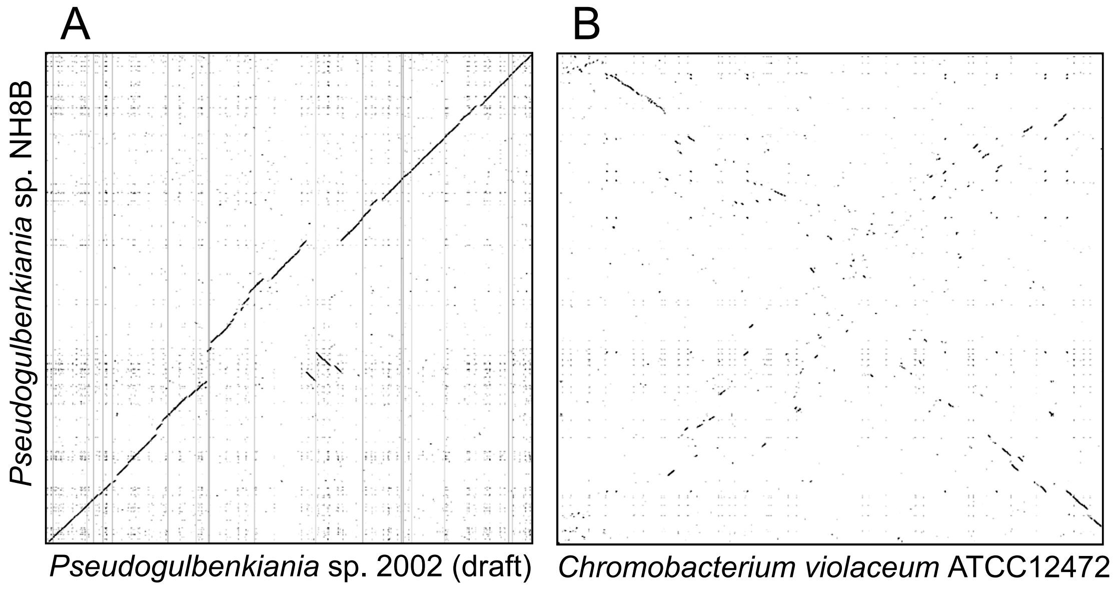

Figure S4. Dot-plot analysis (A) between *Pseudogulbenkiania* sp. strains NH8B and 2002 and (B) between strain NH8B and *Chromobacterium violaceum* ATCC 12472.

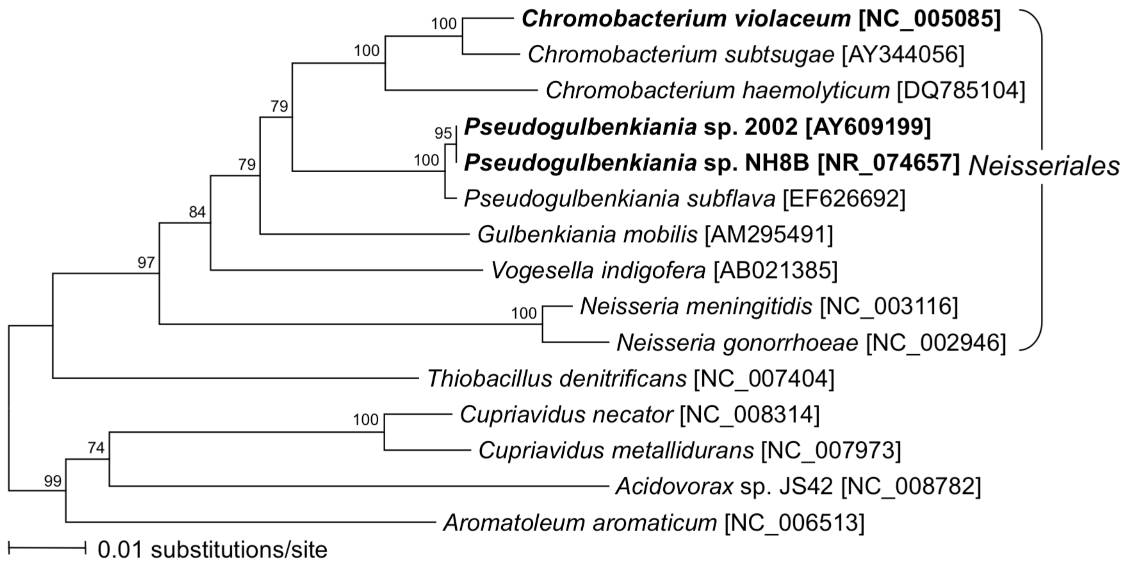

Figure S5. Phylogenetic relationships between strains *Pseudogulbenkiania* sp. strain NH8B, *Pseudogulbenkiania* sp. strain 2002, and some members of the family *Neisseriales* based on the full length 16S rRNA gene sequences. Phylogenetic tree was constructed using maximum likelihood method. The bootstrap values (>70%) from 1,000 replicates are indicated next to the branches. The accession numbers of the reference strains in the DDBJ/EMBL/GenBank databases are indicated in brackets.

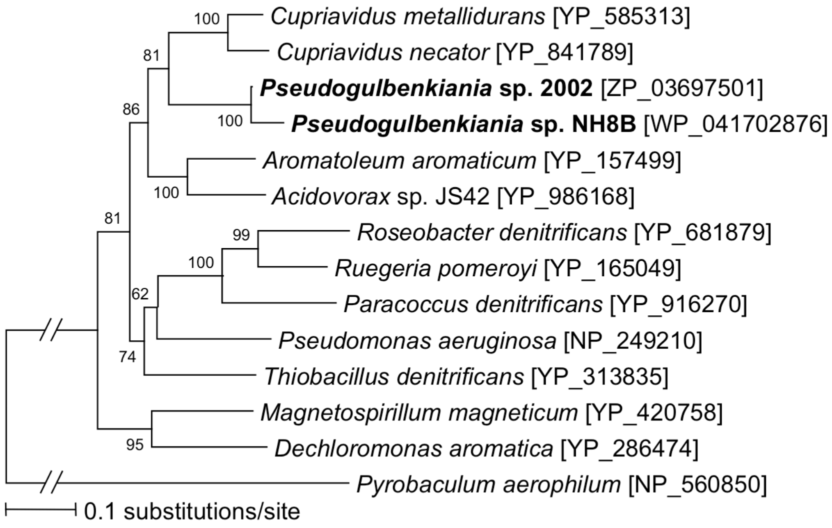

Figure S6. Phylogenetic relationships of the deduced NirS amino acid sequences between strains *Pseudogulbenkiania* sp. strain NH8B and *Pseudogulbenkiania* sp. strain 2002. Phylogenetic tree was constructed using maximum likelihood method. The bootstrap values (>70%) from 1,000 replicates are indicated next to the branches. The accession numbers of the reference strains in the DDBJ/EMBL/GenBank databases are indicated in

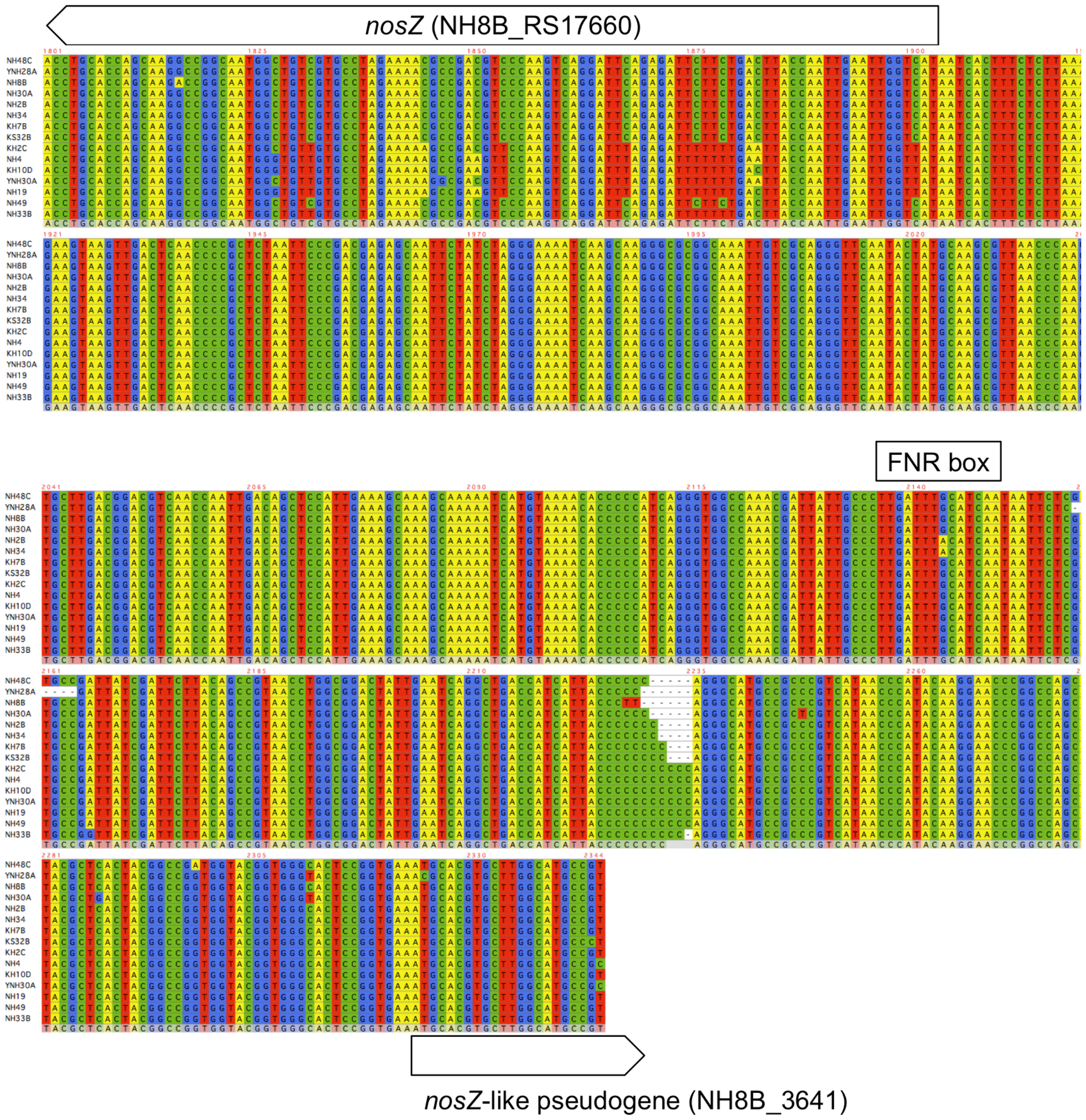

brackets.

Figure S7. Nucleotide sequence alignment of the intergenic region between *nosZ* and *nosZ*-like pseudogene from 15 NH8B-type *Pseudogulbenkiania* sp. strains. Region of the FNR-binding motif (FNR box; TTGAT----ATCAA) is also shown.

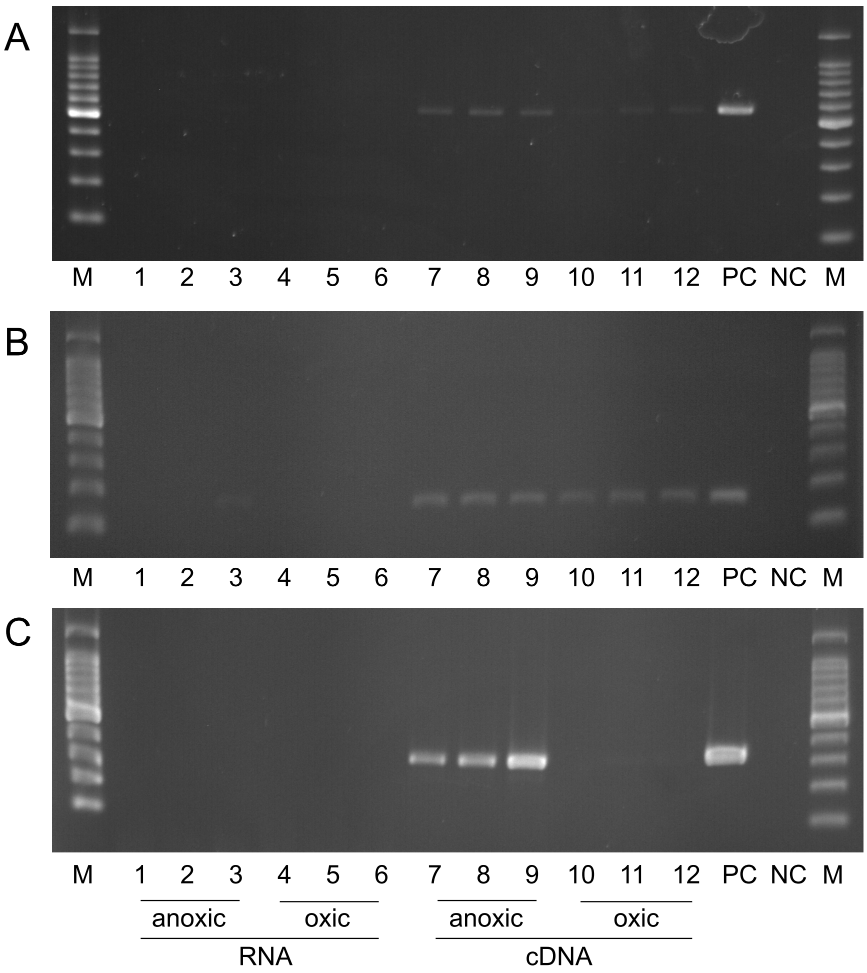

Figure S8. Gel images of the amplicons obtained by reverse transcription PCR done with (A) strain NH8B and NH8B_3826510F and NH8B_3827082R primers, (B) strain NH8B and NH8B_3828317F and NH8B_3829464R primers, and (C) strain 2002 and 2002_323853F and 2002_324186R primers. Legends: 1–3, RNA extracted from cells incubated under anoxic conditions; 4–6, RNA extracted from cells incubated under oxic conditions; 7–9, cDNA synthesized from RNA 1–3, respectively; 10–12, cDNA synthesized from RNA 4–6, respectively; genomic DNA used as a positive control; NC, negative control (nuclease-free water); M, 100-bp DNA ladder.
